# Interocular Symmetry of Macular Ganglion Cell Complex Thickness in Young Chinese Subjects

**DOI:** 10.1371/journal.pone.0159583

**Published:** 2016-07-25

**Authors:** Minwen Zhou, Bing Lu, Jingke Zhao, Qiu Wang, Pengfei Zhang, Xiaodong Sun

**Affiliations:** 1 Department of Ophthalmology, Shanghai First People’s Hospital, School of Medicine, Shanghai JiaoTong University, Shanghai, China; 2 Shanghai Key Laboratory of Fundus Disease, Shanghai, China; Bascom Palmer Eye Institute, University of Miami School of Medicine;, UNITED STATES

## Abstract

**Purpose:**

To report interocular differences in macular ganglion cell complex (mGCC) thickness in young Chinese subjects using RTVue-100 optical coherence tomography (OCT).

**Methods:**

This was an observational, cross-sectional study. The mGCC thickness was measured in 158young Chinese subjects using RTVue-100 OCT. The normal ranges of the interocular differences were determined as falling between the 2.5th and 97.5th percentiles. Right and left eyes were compared using a paired *t* test. Pearson correlation analysis was performed to assess the relationships between mGCC thickness and other potential factors. The relationships between the interocular difference in the average mGCC thickness and the potential factors were evaluated by univariate and multivariate linear regression analysis.

**Results:**

The mean interocular difference in the average, superior, and inferior mGCC thickness were 0.19 ± 2.69 μm, 0.22 ±3.14 μm, and 0.25±3.34 μm, respectively, which were not statistically significant. The 2.5th and 97.5th percentiles of interocular difference for mean average mGCC thickness were -4.82μm and 4.38μm, for superior mGCC thickness, -6.67 μm and 7.04 μm, and for inferior mGCC thickness, -6.75 μm and 6.27 μm. There was a strong correlation between the right and left eyes for all the studied parameters, including spherical equivalent (SE) and axial length (AL). Interocular difference in SE (*p* = 0.007) were independently correlated with the interocular difference in average mGCC thickness.

**Conclusions:**

There was no significant relative interocular difference in mGCC thickness in young Chinese subjects. Interocular difference exceeding the normal limits should be considered significantly asymmetrical, and suggestive of pathology.

## Introduction

Glaucoma is an optic neuropathy characterized by the gradual death of retinal ganglion cells (RGCs) and their axons, leading to visual field loss[1,2].Because of the chronic, imperceptible, and progressive nature of this disease, diagnosis at the asymptomatic stage is difficult for some patients. Thus, timely detection and evaluation of glaucomatous damage through structural and functional analyses are important.

The development of a noninvasive optical imaging technique (OCT) with high-axial-resolution images has enabled clinicians to obtain reproducible measurements of the thicknesses of many layers of the retina[3,4,5]. The RTVue-100 OCT allowed integrated and automatic scanning and measurement of the thickness of the macular ganglion cell complex (mGCC). The mGCC thickness is comprised of the retinal nerve fibre layer (RNFL), retinal ganglion cell (RGC) layers, and the inner plexiform layer, which is directly influenced by glaucomatous ganglion cell loss[6,7]. Thus, the mGCC thickness might be an important indicator of early glaucoma. In fact, several studies have found that the mGCC thickness in glaucoma patients was thinner than in normal subjects and that it was a good diagnostic value for detecting glaucoma[8,9,10].

It has been reported that interocular asymmetry in the optic nerve head and RNFL is an early sign of glaucomatous damage[11,12]. This raises the question of how to define interocular asymmetry in the mGCC thickness and whether it is also an early sign of glaucomatous damage. It would be useful to know the normal range of interocular variation in mGCC thickness and its associated factors.

Juvenile-onset open-angle glaucoma is an uncommon type of primary open-angle glaucoma that usually affects subjects during childhood or early adulthood. However, there is only limited data focusing on the ocular parameters of normal juveniles which attenuates it's value in the detection of juvenile glaucoma. In addition, limited data are currently available regarding interocular symmetry of mGCC thickness in normal populations—especial in juveniles. Thus, the present study measured and assessed the interocular symmetry of mGCC thickness profiles in young Chinese subjects to establish the limits beyond which a clinician should suspect a pathological condition.

## Methods

### Subjects and enrolment criteria

This was an observational, cross-sectional study approved by the Ethical Review Committee of the First People’s Hospital affiliated with Shanghai JiaoTong University. The study abided by the tenets of the Declaration of Helsinki. Written informed consent was obtained from each participant in the study, which took place between June 2015 and July 2015. Eligibility was determined for each subject in a thorough ophthalmic examination, including best-corrected visual acuity (BCVA), slit-lamp examination, intraocular pressure (IOP) measurement, refractive error by optometry (Nidek AR-310A, Japan), spherical equivalent (SE) which was calculated as the sum of the spherical power and half of the cylinder power[13], fundus examination, axial length (AL) measurement (IOL Master; Carl Zeiss Meditec, La Jolla, CA, USA), and central corneal thickness (CCT) measurement. All the subjects completed a questionnaire assessing demographic information and medical history, and underwent anthropometric examination which included heart rate, blood pressure, and body mass index (BMI). Heart rate and blood pressure were measured at the time of the OCT imaging.

The inclusion criteria were BCVA of 20/20 or better, age between 18 and 32 years, normal IOP, good-quality OCT images, and absence of glaucomatous optic neuropathy. The exclusion criteria were any ocular disease except ametropia, history of ocular hypertension or glaucoma, history of ocular surgery, any systemic disease, and inability to tolerate OCT angiography.

### Optical coherence tomography scanning procedure

All subjects were examined with the Fourier-domain OCT system (RTVue-100, Optovue, Fremont, CA, USA) using the scan protocol “GCC” (ganglion cell complex) by an experienced technician blinded to the patient's data. The Fourier-domain OCT imaging was performed as described in previous studies[14,15]. In brief, all the subjects were required to gaze at the fixation target in the OCT machine. The GCC scan protocol was centered 1mm temporal to the fovea and covering a 7 mm ×7 mm area of the central macula. Three average mGCC thickness parameters (superior, inferior, and average) were analyzed. The term “mGCC thickness parameters” refers to the thickness of all macular layers between the internal limiting membrane and the inner plexiform layer in the area above or below the horizontal meridian, or their average. Poor-quality images, defined as those with a signal-strength index less than 40, were excluded[14].

### Statistical analyses

Statistical analyses were performed using SPSS software package (Version 17.0; SPSS Inc., Chicago, IL, USA). For all the tests, a *p* value < 0.05 was considered significant. The interocular differences in mGCC thickness were summarized by their mean, median, 95% confidence interval, and range. The limits of the normal range for interocular differences in mGCC thickness were established as the 2.5th and 97.5th percentiles. The values from the two eyes were compared using the paired *t* test. Bland-Altman plots for interocular mGCC thickness agreement were performed by calculating the difference between right and left eyes plotted against the mean thickness of the two eyes. Horizontal dashed lines were drawn at the 95% limits of agreement, which were defined as the mean difference ±1.96 × standard deviations (SD) of the differences. The correlations between right and left eyes were analyzed by Pearson correlation analysis, which was also performed to evaluate the relationships between the mGCC thickness and IOP at imaging, SE, AL, CCT, systolic ocular perfusion pressure, diastolic ocular perfusion pressure, and mean ocular perfusion pressure in each eye, as well as age, gender, heart rate, systolic blood pressure, diastolic blood pressure and BMI. The relationships between the interocular difference in the average mGCC thickness and the potential factors were evaluated by univariate and multivariate linear regression analysis. Univariate regression analyses were performed separately for each variable; subsequently, variables with a probability value of less than 0.05 were included in the multivariate analysis by a stepwise method.

## Results

### Demographics and clinical characteristics of the subjects

Of all the recruited subjects for this study, one subject was excluded because of a glaucoma diagnosis and another subject was excluded after a finding of choroidal neovascularization. Three more subjects were excluded because they did not finish the OCT examination. The final sample set consisted of 158 subjects (316 eyes). The percentage of females was significantly higher than males. The mean age of the subjects was 25.95 ± 1.78 years. SE ranged widely, from 3.75 diopters (D) to -10.375 D, with a mean of -3.78 ± 2.54 D. The range of AL was between 21.31 to 28.14mm (mean 24.95 ± 1.18mm). The mean IOP at imaging was 16.34 ± 2.71 mmHg, and the mean central corneal thickness was 538.28 ± 33.97μm. Demographic and baseline clinical characteristics are summarized in Table 1.

**Table 1:** Clinical characteristics in study subjects.

Variables	Mean ± SD
No. of subjects (No. of eyes)	158(316)
Mean age, y	25.95± 1.78
Gender (male/female)	94/64
IOP at imaging, mm Hg	16.34± 2.71
Spherical equivalent, D	-3.78± 2.54
Axial length, mm	24.95 ± 1.18
CCT, μm	538.28± 33.97
SBP, mmHg	115.43± 11.98
DBP, mmHg	68.78± 8.10
Systolic OPP, mmHg^1^	99.09± 12.11
Diastolic OPP, mmHg^2^	52.43± 8.43
Mean OPP, mmHg^3^	67.98± 8.55
Heart rate, BPM	78.19± 11.86
BMI, kg/m^2^	21.28± 2.58

SD: standard deviation; IOP: intraocular pressure; D: diopter; CCT: central corneal thickness; SBP: systolic blood pressure; DBP: diastolic blood pressure; OPP: ocular perfusion pressure BPM: beats per minute; BMI: body mass index

^1, 2^: Calculated as the differential pressure between diastolic or systolic blood pressure and IOP.

^3^: Calculated as the differential pressure between mean BP and IOP (mean BP: DBP+1/3*(SBP -DBP)).

### mGCC thickness

The mean superior mGCC thickness and inferior mGCC thickness were 98.43± 5.70μm and 98.00± 5.79μm, respectively, with a significant difference. The mean average mGCC thickness of all eyes was 98.16 ± 5.53μm. For the right eyes, the mean average mGCC thickness was 98.26 ± 5.54, and for the left, 98.07 ± 5.54 μm, with a mean difference of 0.19 ± 2.69 μm (*p* = 0.381). The mean superior mGCC thickness in the right eye (98.54 ±5.64 μm) was 0.22 ±3.14 μm thicker than in the left eye (98.32 ±5.79 μm); the difference was not significant (*p* = 0.385). Similarly, the differences were not statistically significant for the inferior mGCC thickness between the right and the left eyes (*p* = 0.343). The interocular differences in the mGCC thickness between right and left eyes are listed in Table 2.

**Table 2:** Macular GCC measurements in right and left eyes of Chinese young subjects and interocular differences (right eye minus left eye).

Variables	Right Eye (μm)		Left Eye (μm)		Interocular Difference (Right–Eye) (μm)		Interocular Difference (Right–Eye) (μm)	
	Mean (SD)	Median (Min, Max)	Mean (SD)	Median (Min, Max)	Mean (SD)	Median (Min, Max)	95% CI	*P*
mGCC superior	98.54(5.64)	98.285(81.98,118.10)	98.32(5.79)	98.300(85.51,115.63)	0.22(3.14)	0.075 (-9.30,14.77)	-0.28, 0.71	0.385
mGCC inferior	98.13(5.92)	98.020(78.89,118.70)	97.87(5.67)	97.960(82.58,113.38)	0.25(3.34)	0.245 (-12.30, 23.15)	-0.28, 0.78	0.343
mGCC average	98.26(5.54)	98.050(80.43,118.29)	98.07(5.54)	98.090(84.67,114.13)	0.19(2.69)	0.380 (-8.56, 19.16)	-0.23, 0.61	0.381

mGCC: macular ganglion cell complex; SD: standard deviation; CI: confidence interval

**Fig 1:**
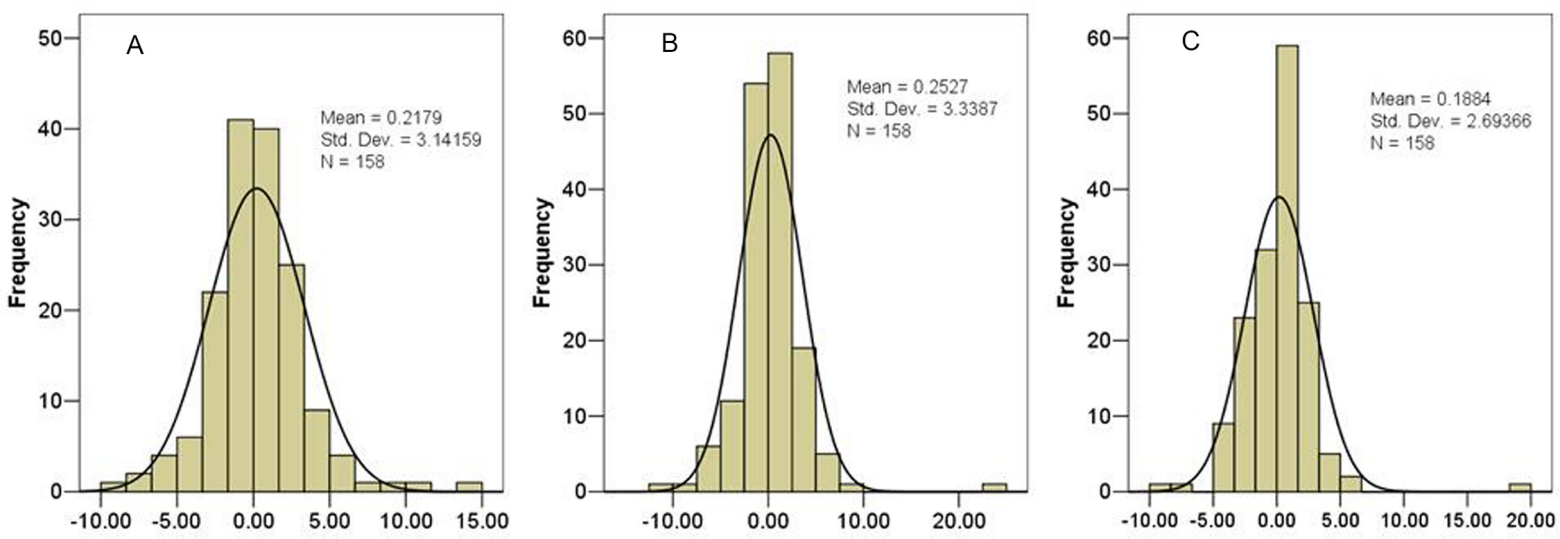
**Graph showing the frequency distribution of mean interocular difference (right eye minus left eye) in superior (A), inferior (B), and average (C) mGCC thickness**.

The percentile distributions of the interocular asymmetry in mGCC thickness are displayed in Table 3, and the mean distribution of interocular differences in the average, superior, and inferior mGCC thickness is shown in Fig 1. The 2.5th and 97.5th percentiles of interocular difference tolerance limits for mean average mGCC thickness were -4.82 μm and 4.38 μm, in superior mGCC thickness-6.67 μm and 7.04 μm, and in inferior mGCC thickness, -6.75 μm and 6.27 μm.

**Table 3:** Percentile distribution of interocular differences (right eye minus left eye).

Variables	Percentile								
	2.5	5	10	25	50	75	90	95	97.5
mGCC superior	-6.67	-4.86	-3.23	-1.51	0.08	1.70	3.66	5.03	7.04
mGCC inferior	-6.75	-5.33	-2.86	-1.11	0.25	1.63	3.42	4.71	6.27
mGCC average	-4.82	-4.10	-2.71	-1.23	0.38	1.45	2.54	3.58	4.38

mGCC: macular ganglion cell complex

**Fig 2:**
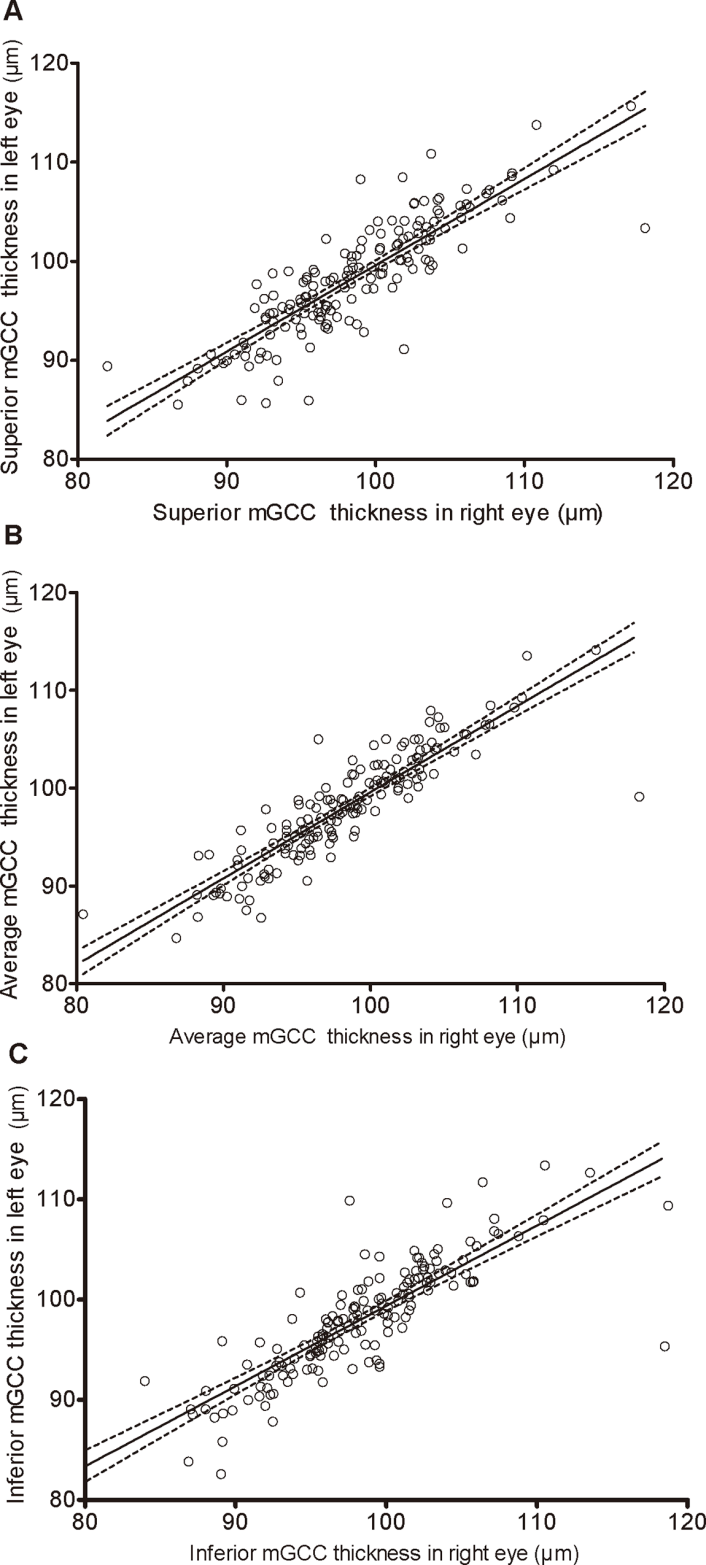
**Scatterplots showing relationship between superior (A), average (B), and inferior (C) mGCC thickness in right eye and in left eye**.

**Fig 3:**
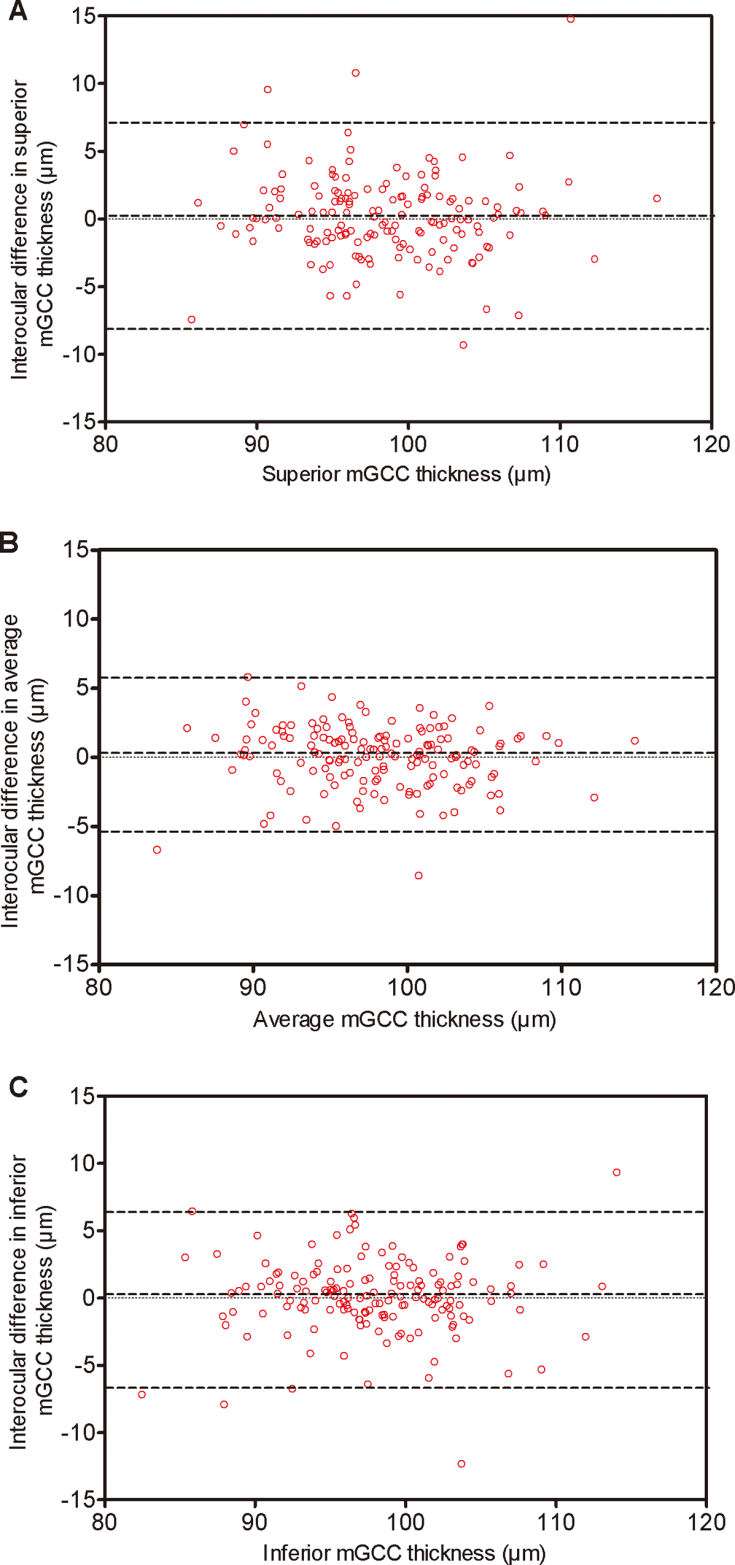
Bland–Altman plots for mGCC thickness of each eye. Dotted lines delineate 95% confidence limits of agreement. There was no specific tendency to cause the difference between right and left eyes. A: superior mGCC thickness. B: average mGCC thickness. C: inferior mGCC thickness.

To look at the data from an interocular agreement perspective, we performed a Pearson correlation analysis, and the detailed results are displayed in Table 4 and Fig 2. The correlation coefficient of the association between the average mGCC thickness in the right and left eyes was *r* = 0.882, indicating a high interocular symmetry. The right and left eyes were also well-correlated at the superior mGCC thickness (*r* = 0.849; *p*<0.001) and the inferior (*r* = 0.835; *p*<0.001). The Bland-Altman plot showed a good agreement between fellow eyes in average, superior, and inferior mGCC thickness (Fig 3).

**Table 4:** Correlations of corresponding loci of mGCC thickness between the right and left eyes.

Correlation	*r*	*P*
Right eye mGCC superior- Left eye mGCC superior	0.849	<0.001
Right eye mGCC inferior- Left eye mGCC inferior	0.835	<0.001
Right eye mGCC average- Left eye mGCC average	0.882	<0.001

mGCC: macular ganglion cell complex

### Factors influencing the mGCC thickness

Pearson correlation analysis was performed to evaluate the relationships between the mGCC thickness and potential factors. The result showed that strong positive correlations were found between average (right eye: *r* = 0.319, *p*<0 .001; left eye: *r* = 0.393, *p*<0 .001), superior (right eye: *r* = 0.269, *p* = 0 .001; left eye: *r* = 0.362, *p*<0 .001), inferior (right eye: *r* = 0.322, *p*<0 .001; left eye: *r* = 0.393, *p*<0 .001) mGCC thickness and SE. Increasing AL was associated with a decrease in average, superior, and inferior mGCC thickness in both eyes. The detailed correlation analysis was summarized in Table 5.

**Table 5:** Pearson correlation were calculated for variation in mGCC thickness in right and left eyes.

	Right eye						Left eye					
	mGCC superior		mGCC inferior		mGCC average		mGCC superior		mGCC inferior		mGCC average	
	*r*	*P*	*r*	*P*	*r*	*P*	*r*	*P*	*r*	*P*	*r*	*P*
Age, y	-0.009	0.916	-0.058	0.470	-0.028	0.731	-0.105	0.191	-0.118	0.139	-0.112	0.160
Gender (male/female)	-0.034	0.667	0.044	0.587	0.005	0.949	0.021	0.792	0.020	0.798	0.026	0.750
IOP at imaging, mm Hg	-0.045	0.574	-0.050	0.531	-0.057	0.476	0.057	0.474	0.029	0.714	0.051	0.527
Spherical equivalent, D	0.269	0.001	0.322	<0.001	0.319	<0.001	0.362	<0.001	0.393	<0.001	0.393	<0.001
Axial length, mm	-0.304	<0.001	-0.360	<0.001	-0.353	<0.001	-0.363	<0.001	-0.390	<0.001	-0.395	<0.001
CCT, μm	0.068	0.396	0.048	0.545	0.059	0.460	0.089	0.267	0.121	0.129	0.109	0.171
SBP, mmHg	0.077	0.334	0.124	0.121	0.107	0.183	0.118	0.139	0.071	0.378	0.094	0.242
DBP, mmHg	0.094	0.241	0.110	0.170	0.100	0.211	0.116	0.147	0.063	0.434	0.088	0.273
Systolic OPP, mmHg^1^	0.087	0.279	0.134	0.093	0.118	0.139	0.104	0.194	0.063	0.430	0.081	0.311
Diastolic OPP,mmHg^2^	0.104	0.193	0.121	0.130	0.114	0.154	0.093	0.245	0.051	0.525	0.068	0.394
Mean OPP, mmHg^3^	0.110	0.169	0.143	0.073	0.131	0.100	0.111	0.167	0.064	0.426	0.084	0.296
Heart rate, BPM	-0.005	0.954	0.024	0.765	0.005	0.947	0.005	0.952	0.019	0.810	0.013	0.874
BMI, kg/m^2^	-0.043	0.593	-0.008	0.916	-0.015	0.847	0.015	0.847	-0.013	0.869	0.000	1.000

mGCC: macular ganglion cell complex; IOP: intraocular pressure; D: diopter; CCT: central corneal thickness; SBP: systolic blood pressure; DBP: diastolic blood pressure; OPP: ocular perfusion pressure; BPM: beats per minute; BMI: body mass index

^1, 2^: Calculated as the differential pressure between diastolic or systolic blood pressure and IOP.

^3^: Calculated as the differential pressure between mean BP and IOP (mean BP:DBP+1/3*(SBP-DBP)).

### Factors influencing interocular differences in average mGCC thickness

**Fig 4:**
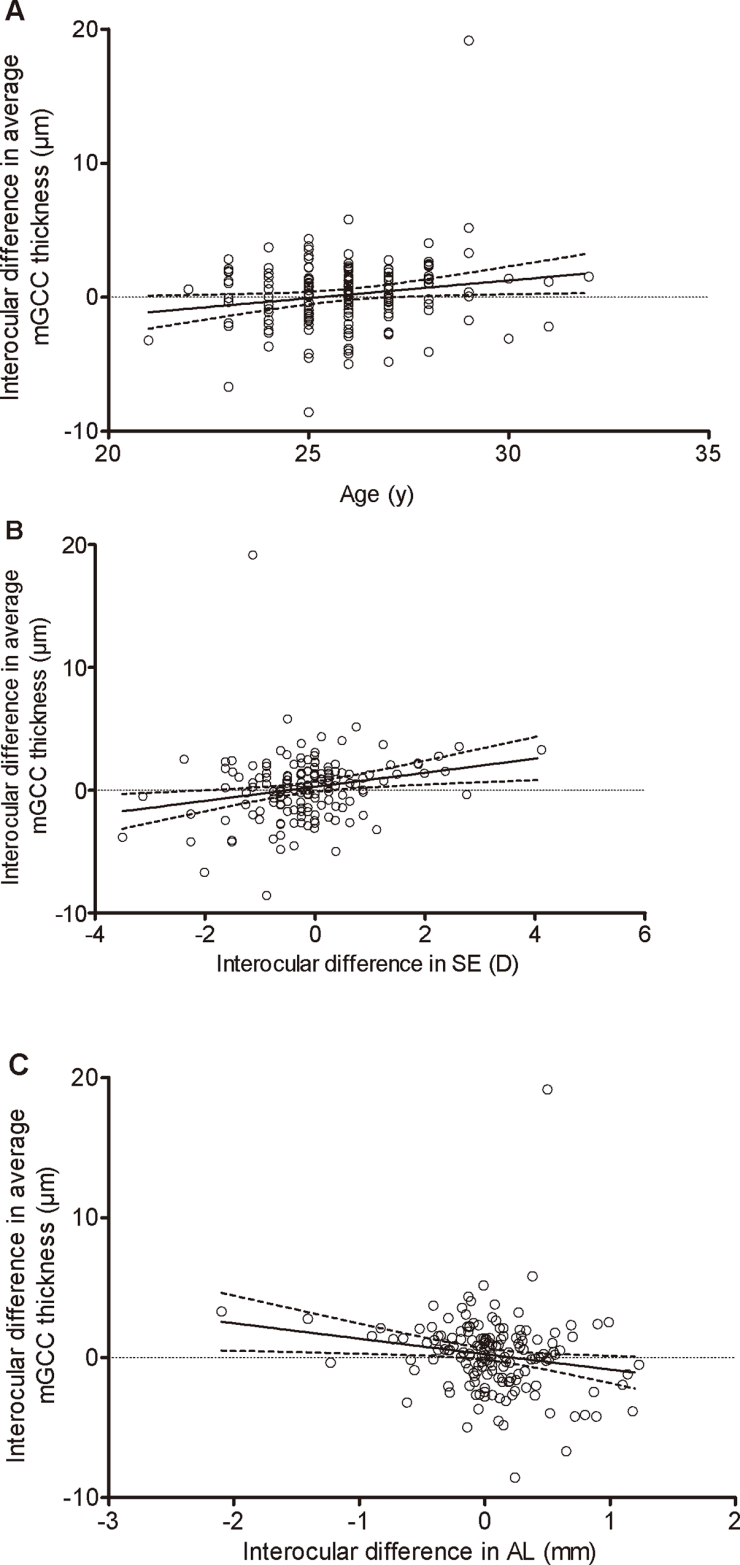
**Scatterplot showing linear regression analysis between age (A), interocular difference in SE (B), and interocular difference in AL (C) and the interocular difference in the average mGCC thickness**.

With regard to interocular differences of average mGCC thickness, univariate and multivariate regression analyses were performed to determine the associated factors. The interocular difference in the average mGCC thickness was significantly correlated with age (*p* = 0.029), interocular difference of the SE (*p* = 0.007), and interocular difference of the AL (*p* = 0.022). Subsequently, variables with a probability value of less than 0.05 were included in the multivariate analysis by a stepwise method, and the multiple regression analysis showed that interocular difference in SE (*p* = 0.007) were independently correlated with the interocular difference in average mGCC thickness. Table 6 displays the detailed result of regression analyses of interocular differences of average mGCC thickness. The scatter plots are displayed in Fig 4.

**Table 6:** Factors associated with the interocular difference in the average mGCC thickness in Chinese young subjects.

	Average mGCC thickness			
Variables	Univariate Analysis		Multivariate Analysis	
	Beta (95% CI)	*P*	Beta (95% CI)	*P*
Age, y	0.174(0.028,0.500)	0.029		
Gender (male/female)	-0.042(-1.062,0.589)	0.615		
Interocular difference (right eye minus left eye)					
IOP at imaging, mm Hg	0.047(-0.164,0.302)	0.559		
Spherical equivalent, D	0.214(0.160,0.987)	0.007	0.214(0.160,0.987)	0.007
Axial length, mm	-0.182(-2.047,-0.159)	0.022		
CCT, μm	-0.027(-0.054,0.039)	0.740		

mGCC: macular ganglion cell complex; CI: confidence interval; IOP: intraocular pressure; D: diopter; CCT: central corneal thickness

## Discussion

It is well-known that the macula plays an important role in visual function and that it has attracted a large number of researchers. The mGCC, which includes the RNFL, RGC layers, and inner plexiform layer, has been the focus for diagnosis of glaucoma[16,17]. The mGCC thickness is comparable to the RNFL thickness as a diagnostic indicator of glaucoma [15]. Therefore, early detection of mGCC damage is useful for the management of glaucoma. With the recent technological enhancements in OCT, clinicians can detect mGCC damage in a more quantitatively reliable manner.

Generally, glaucoma is a bilateral disease; however, it often shows asymmetric characteristics. Therefore, determining the normal cutoff values for interocular differences of mGCC would help detect glaucoma in a more timely fashion. If the interocular asymmetry exceeds normal limits, it should alert the clinician to possible glaucomatous damage and be an indicator for further examination. Thus, the aim of this study was to comprehend the interocular symmetry of mGCC thickness in young Chinese subjects.

To the best of our knowledge, our study is the first to investigate the interocular symmetry of mGCC thickness in young subjects using the RTVue-100 OCT. When the right and left eyes were compared, moderate symmetry was found to exist in the mGCC thickness. Bland-Altman plots showed that the difference between the right and left eyes was evenly dispersed around the mean and that the interocular differences at any of the macular regions (superior, inferior, and average) were not statistically significant. Additionally, the correlation analysis displayed a high correlation between the superior, inferior, and average mGCC thickness measurements of fellow eyes. The correlation coefficients were all above 0.8, which implied a high correlation and further confirmed the symmetry in bilateral eyes. Generally speaking, values corresponding to the central 95 percentiles should be considered normal. Therefore, values below the 2.5th and above the 97.5th percentiles represent outliers and could indicate pathology. In the present study, the cut-off limits for normal interocular differences (between the 2.5th and 97.5th percentiles of normative data) of mGCC thickness were -6.67 and 7.04 μm, -6.75 and 6.27 μm, and -4.82 and 4.38 μm for the superior, inferior, and average measurements, respectively. The clinical significance is that any interocular asymmetry which exceeds these cut-offs could indicate potential disease and it should call for our attention to perform a further examination to determine whether they have any eye diseases. It might be worth following up to see whether any of them could develop glaucoma in the future. According to these findings, the interocular symmetry of mGCC thickness measured by OCT can help to distinguish normal from glaucomatous eyes.

A Pearson correlation analysis was performed to determine the factors associated with mGCC thickness in both the right and left eyes. The results showed that mGCC thickness in both eyes was significantly correlated with AL and SE. Zhao et al.[18]and Hirasawa et al.[19]reported thicker mGCCs in adult eyes with shorter AL. Zhao et al.[20]also found that mGCC thicknesses were significantly associated with SE. This is consistent with our findings. It is worth noting that in the present study, the correlation coefficients of the superior, inferior, and average mGCC thickness with SE and AL were quite similar in both eyes. This also implied the interocular symmetry of mGCC.

In the past few years, numerous studies have focused on the interocular difference in RNFL thickness as an indicator of glaucoma. For example, Lee et al.[21]reported statistically significant interocular differences in RNFL thickness. Park et al.[22], using the Stratus OCT, found that the nasal and temporal sectors of the RNFL were significantly thicker in the right eye than in the left. Hwang et al.[23]demonstrated that significant interocular differences in RNFL thickness exist in healthy eyes. Unlike the results of previous studies in which RNFL thickness was assessed, the present study assessed the average, superior, and inferior mGCC thickness and showed no statistically significant interocular asymmetry. These two parameters could be diagnostic indicators of glaucoma. In addition, the GCC layer included the RNFL; however, the two parameters displayed different symmetry characteristics. Possible explanations for this difference could be that it is region-specific. We speculated that macular parameters might be more symmetrical than those of the RNFL. In fact, this phenomenon was found in other studies. Altemiret al.[24]assessed interocular symmetry in RNFL thickness and macular thickness and found that macular thickness was symmetrical but RNFL thickness was not. Chen et al.[25]also assessed interocular symmetry of macular choroidal thickness at the fovea and found it to be highly symmetrical.

When assessing the correlated factors of interocular difference in the average mGCC thickness, in univariate analysis, the interocular difference in the average mGCC thickness was significantly correlated with age, interocular differences of SE, and interocular differences of AL. In a multivariate analysis, we found that the interocular difference in the average mGCC thickness increased with the increased interocular differences in SE. These findings suggest that interocular differences in SE should be taken into account when evaluating interocular symmetry in the mGCC thickness.

The strengths of this study were first, the young age of the subjects, with no confounding factors of systemic diseases or ocular pathology, and that they could fixate well on the target during the imaging. Second, the subjects received a comprehensive ocular examination with detailed biometric measurements. Third, this was the first study to investigate the interocular symmetry of mGCC thickness with a relatively large sample size.

The limitations of our experiment that should be considered when discussing the results are first, that the included subjects were all young adults, resulting in a narrow age range and lack of data from older subjects. However, these data will informative in constructing normative profiles for clinical and research purposes in those Juvenile-onset open angle glaucoma diagnosis. Second, the present study only included Han Chinese subjects and thus lacked ethnic variety. Third, our study recruited only healthy subjects; it therefore lacked data regarding the interocular symmetry of mGCC thickness in the glaucoma population.

In summary, our study demonstrated no significant interocular asymmetry in the average, superior, and inferior mGCC thicknesses in young Chinese subjects, and the cut-off limits for normal interocular differences of average mGCC thickness were -4.74μm and 4.27μm. Interocular differences exceeding these values should be considered significantly asymmetrical and suggestive of pathology.

## Supporting Information

S1 Dataset Data of the Experiment. (XLS)
